# Mouthwitch: A Novel Head Mount Type Hands-Free Input Device that Uses the Movement of the Temple to Control a Camera

**DOI:** 10.3390/s18072273

**Published:** 2018-07-13

**Authors:** Kazuhiro Taniguchi, Atsushi Nishikawa

**Affiliations:** 1 Graduate School of Information Sciences, Hiroshima City University, 3-4-1 Ozukahigashi, Asaminami-ku, Hiroshima 731-3194, Japan; 2 Faculty of Textile Science and Technology, Shinshu University, 3-15-1 Tokida, Ueda, Nagano 386-8567, Japan; nishikawa@shinshu-u.ac.jp; 3 Division of Biological and Medical Fibers, Institute for Fiber Engineering (IFES), Interdisciplinary Cluster for Cutting Edge Research (ICCER), Shinshu University, 3-15-1 Tokida, Ueda, Nagano 386-8567, Japan

**Keywords:** temple, mouth, non-invasive, optical measurement, hand-free controller, head mount camera

## Abstract

We have developed an interface (mouthwitch) for a head-mounted type camera with which pictures can be taken with a head-mounted camera, hands-free, simply by “opening your mouth continuously for approximately one second and then closing it again”. This mouthwitch uses a sensor equipped with an LED and photo transistor on the temple to optically measure the changes in the form of the temple that occur when the mouth is opened and closed. Eight test subjects (males and females aged between 21 and 44 years old) performed evaluation tests using this mouthwitch when resting, speaking, chewing, walking, and running. The results showed that all test subjects were able to open and close the mouth, and the measurement results pertaining to the temple shape changes that occurred at this time were highly reproducible. Additionally, the average value for *accuracy* obtained for the eight test subjects through the verification tests was 100% when resting, chewing, or walking, and 99.8% when speaking or running. Similarly, the average values for *precision* were 100% for all items, and the average values for *recall* were 100% when resting or chewing, 98.8% when speaking, 97.5% when walking, and 87.5% when running.

## 1. Introduction

With the spread of social networking services, such as Instagram and Twitter, in recent years, the need to take photographs on a daily basis has increased [1,2,3,4,5]. Additionally, lifelogging, in which photographs are taken automatically, is attracting attention in the memory rehabilitation field [6,7,8]. We use smartphones and digital cameras when taking photographs. If you want to use your smartphone or camera to take photographs, without missing important moments, you need to be ready to take a picture at any time. In other words, it is necessary to always be equipped with a smartphone or a camera just as you would be if you were a cameraman. Realistically, it is difficult to be ready with your smartphone or camera at any time. If you use eyeglass-type terminals equipped with cameras, such as the SmartEyeglass SED-E1 (Sony Corp., Tokyo, Japan) [9], you will not have to be always be prepared; however, since the shutter needs to be pressed manually, you will always have to leave your hands available to operate the shutter. Eyeglass-type terminals, such as GLΛSS (X Development LLC., CA, USA) [10,11,12] etc., using voice recognition technology, have functions that enable you to take photographs with just your voice, without using your hands. However, voice technology has a disadvantage because the accuracy of the recognition is influenced by surrounding noise, and only registered languages can be used. Additionally, in locations where silence needs to be maintained, photographs cannot be taken with due consideration of courtesy. Methods other than voice recognition include operating the shutter by blinking, and the development of devices [13] that can be attached to glasses; however, if you close your eyes, it will be difficult to capture an important scene that you want to preserve while looking at it.

The SenseCam is an effective device for use in lifelogging [14,15]. It is a lightweight, non-invasive digital camera, and is used by being hung around one’s neck like a pendant. It uses light, heat, or time as photograph triggers, and automatically takes pictures independently (without the user operating by hand) of the user’s will. The SenseCam, with its wide-angle lens can capture pictures over a wide range. Using this, lifelogging is capable of building autobiographical memories for improving memory, which is useful for memory rehabilitation in the elderly and those with mild forms of Alzheimer’s disease [6,7,8].

The lightweight, non-invasive wearable camera is suited for taking photographs for Instagram, Twitter, and lifelogging. Because there are countries, such as Japan, where taking photographs of people without their permission is punishable by law, it is important, particularly in the case of photographs that will be published on the Net, to ensure that additional people are not photographed. When using wearable cameras for Instagram or Twitter photography, it is important to be able to take pictures when the user wants to take pictures by exercising their free will, and to be able to take pictures close to the scenes the user is looking at (only scenes the user intended). A mechanism by which surrounding people can be aware pictures being taken is also required to prevent non-consensual photography.

To match the landscape seen by your eyes and the captured photographs and videos, it is necessary to attach the camera close to your eyes (i.e., it is necessary to install the camera in your own line of sight). Methods of realizing this include a method of embedding a camera in an eyeglass-type terminal [9,10,11,12] and a method in which the eyeglass seal is equipped with a camera [13]. However, those people who are already wearing glasses would need to insert a lens at a degree matching their own eyesight into the camera-embedded glasses, and this would increase the cost of installation. Alternatively, if you are attaching a camera to the glasses that you are currently using, the extent to which the shape of the camera attachment section and the temples of the spectacles match greatly affects the stability of the camera, and thus directly influences the quality of the picture taken.

Therefore, we developed a compact, lightweight wearable terminal that can take pictures and video, by the user’s volition, from a position and angle close to the eyeline of the user. This wearable terminal has a mechanism by which it can both take pictures hands-free and it is easy for surrounding people to know that pictures are being taken. We have attached a small camera to the head of the participant and developed a head-mounted camera that can capture photographs and video, hands-free, by simply opening and closing your mouth. Opening and closing the mouth is an action that most people can perform simply, even if they cannot move their arms and legs freely or speak the specific language registered in the device, and the extension and contraction of the temporalis that accompanies the opening and closing of the mouth can be freely done by most people. Activating the shutter “press button” by moving the temporal muscle changes a freely movable part of the body for the button, and this can be linked reliably to the shutter operation. Measuring the opening and closing of the mouth is achieved by attaching a sensor for measuring the movement of the temporalis, which is one of the muscles in the chin, to the head of the participant. More specifically, shape changes near the temple that move with the extension and contraction of the temporalis, are measured by the sensor. Of methods for measuring the extension and contraction of the temporalis, it is conceivable to use muscle potential, but in order to make the device compact and lightweight, we used optical sensors for the biosensor (details are explained in Section 2). The temporalis is one of the muscles closest in position to the head-mounted camera, so by using the temporalis for the shutter operation, the camera and biosensor can be concentrated in one area of the body, thus making the device more compact.

This device is in a head-mounted form that does not interfere with the wearing of glasses, so it can be worn by both those who use as well as those who do not use glasses on a daily basis. Additionally, the opening and closing of the mouth to operate the shutter is distinguished from opening and closing related to speaking, eating, and breathing in everyday life, by using the operation of “opening your mouth continuously for approximately one second and then closing it again”, which is rarely encountered during everyday life. As the temporalis is also moved during the chewing operation, it is considered possible that the shutter operation would be activated by “clenching the back teeth for one second” [16,17], but in this study we adopted the use of opening and closing of the mouth, which could be used even by people with bad teeth. Additionally, opening and closing of the mouth can signify to those around you that you are taking a picture. In other words, in case the subject of the photograph is a human, it is possible to communicate the timing of the shutter to the subject, and this also prevents voyeuristic photos. Of course, in addition to communicating the timing of the shutter to the subject by opening your mouth with this method, you can also use your voice. In this study, we aim to realize operation with a small-scale system configuration, compared to voice operations, which require a microphone and advanced voice recognition software. Moreover, not only can this method be used in the same way as voice recognition, by people who cannot use their hands and feet freely, but because it is not restricted by the language used, it can be used by a large number of people. Thus far, we have succeeded in the development, as a hands-free switching device, of an earphone-type device known as the earable TEMPO [18,19,20,21,22]. The earable TEMPO, by the user moving their tongue, can start/stop a portable music player “without anyone noticing”. As earphones are a compulsory item for a portable music player, if we use the earable TEMPO, it will be possible to operate the portable music player with a minimal amount of system configuration. This earable TEMPO can also be applied to the shutter operation, but with a head-mounted camera, building the device for operating the shutter into the equipment being worn means that the system configuration can be reduced. Moreover, rather than the movement of the tongue, which nobody would notice, opening and closing one’s mouth, which those around you would be aware of, is considered more suitable as a shutter operation.

In this paper, as part of the research and development for a head-mounted camera that can operate a shutter, hands-free, by simply opening and closing the mouth in one-second intervals, we examine the interface (mouthwitch) for measuring the opening and closing of the mouth for a one-second interval, and correctly operating the ON/OFF switch and pressing the button, based on these measurement results. The results of the performance evaluation tests will also be discussed. 

The mouthwitch is named after the meaning of the device switch for opening and closing the mouth (Mouth + switch). Its name is also from the fact you can operate the device without using your hands, simply with a movement of the mouth, looks as if a witch was using magic (Mouth + witch).

## 2. Materials and Methods

### 2.1. Hardware

**Figure 1:**
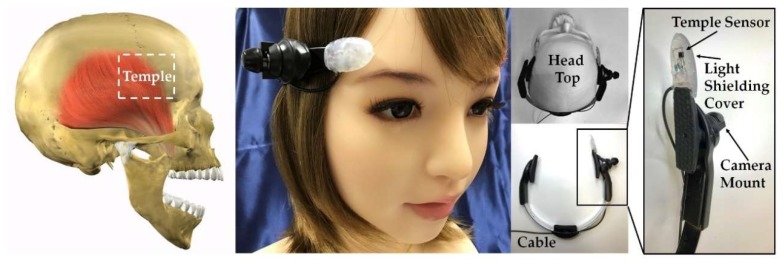
Mouthwitch external appearance and attachment diagram. When performing the operation of opening and closing the mouth, the temporal muscle, which is one of the muscles that moves the chin, is extended and contracted. The mouthwitch, through the right temporal muscle movement, measures the movement of the skin near the right temple using an optical sensor (temple sensor). This temple sensor is equipped with an infrared LED and phototransistor, and the movement of the temporal muscle is measured by infrared light being projected to the skin near the right temple, and this reflected light is received by the phototransistor. This temple sensor is attached to the head-mounted equipment shown in Figure 2, to measure the movement of the skin near the right temple of the test subject. The temple sensor is covered using a light-shielding cover to prevent excess environmental light (direct sunlight, etc.) from entering the phototransistor. The weight of the head-mounted equipment is 82 g. The cable stretching from the head-mounted equipment is connected to a microprocessor for processing the signal results and outputting the shutter operation signal, speakers, and a power supply.

**Figure 2:**
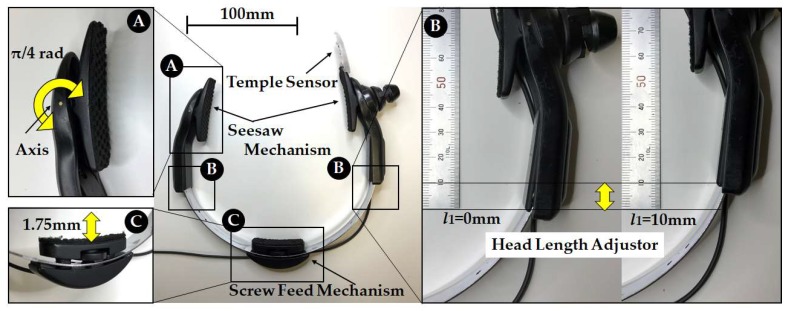
Head-mounted equipment size adjustment mechanism: This equipment is fixed to the head of the participant with the camera and sensor (temple sensor) for measuring the movement of the right temporal region to the three parts of the left and right temporal region and occipital region. This is adjusted to the shape of the test subject’s head using the three areas of the temporal region ((**A**) Seesaw Mechanism), head length ((**B**) Head Length Adjustor), and occipital region ((**C**) Screw Feed Mechanism).

**Figure 3:**
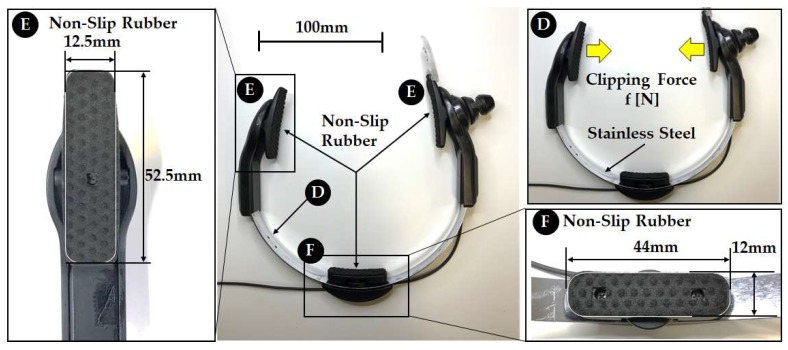
Head-mounted equipment non-slip mechanism: To reliably fix the temple sensor and camera to the head of the participant, the left and right temporal regions are clipped using a C-shaped elastic section (**D**), preventing shaking of the camera and sensor due to motion. A Non-Slip Rubber (**E**,**F**) has been attached to prevent slipping of the hair or skin, and this, in turn, prevents misalignment of the equipment due to bodily movement.

**Figure 4:**
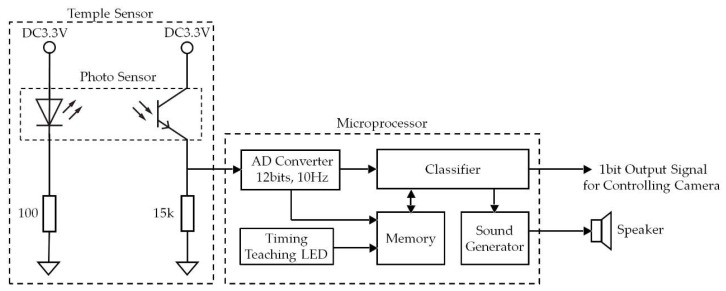
Mouthwitch block diagram. Mouthwitch measures the movement of the skin on the right temple caused by opening and closing the mouth; these measured values are converted from analog to digital signals, and based on the results, when the classifier judges that the test subject was “opening their mouth continuously for approximately one second and then closing it again”, it outputs a 1-bit shutter operation signal to the camera. The classifier obtains a correlation between the TMC ground truth for the test subject stored in memory in advance, and the measurement results, and if this is 0.9 or higher, judges that “the test subject has performed TMC”, a shutter sound (“Do” sound) generated by the sound generator is output from the speakers, and the test subject is able to confirm whether the shutter operation has been performed correctly based on this shutter sound.

Mouthwitch measures the extension and contraction (movement of the skin around the temple) of the temporal muscle when opening and closing the mouth. When it judges that you are “opening your mouth continuously for approximately one second and then closing it again”, it sends a 1-bit output signal to operate the camera shutter. This temple and mouth movement for operating the camera shutter is referred to as TMC (Temple and Mouth movement for Camera operation). The external appearance of the mouthwitch prototyped in this study and a diagram showing its attachment are given in Figure 1, and the equipment (head-mounting equipment) for fixing the sensor and camera to the head of the participant to measure the movement of the temple are shown in Figure 2 and Figure 3. Figure 4 shows the mouthwitch block diagram. In this study, the three important development items are the “head-mounting equipment”, the “method of measuring the movement of the skin near the temple”, and the “algorithm classifying (searching for) TMC from these measurement results”.

**Figure 5:**
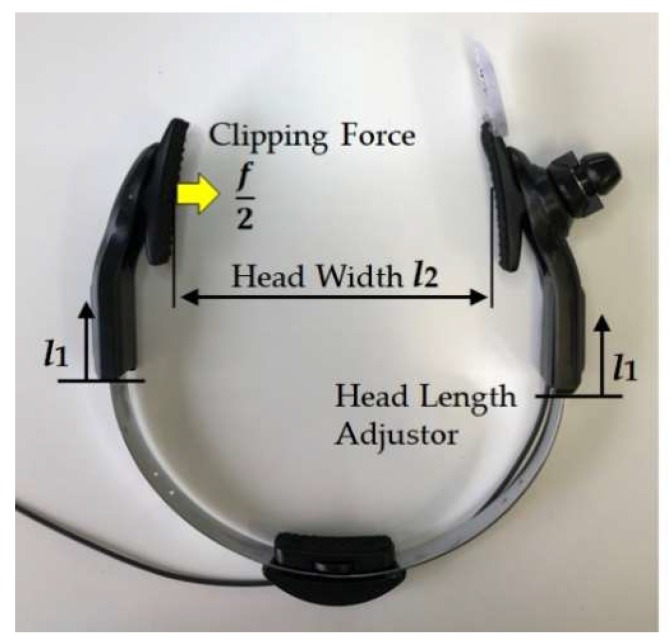
Measurement of the pinching force f of the head mounted equipment: The Clipping Force *f*/2 N when the head-mounted equipment Head Length Adjustor l1 is changed is set to 0 and 10 mm, and the Head Width l2 is changed in increments of 10 mm between 120 mm and 180 mm is measured using Digital Force Gauge.

Mouthwitch, as shown in Figure 1, is head-mounted equipment that is shaped like the letter C, linking the right temporal region, occipital region, and left temporal region. The right temporal region of the mouthwitch has a camera attachment section that can be equipped with a compact and lightweight camera, and this is equipped with a sensor (temple sensor) that measures the movement of the temple. The head-mounted equipment, as shown in Figure 2, can be moved passively in the range of π/4 rad, with the surface touching the temporalis centering on an axis, using a see-saw mechanism (Figure 2A). Additionally, on this surface, because a rubber plate is mounted to prevent hair, skin, and the equipment from slipping, the temple sensor or camera can be firmly secured in the temporal region (Figure 3E). Furthermore, even in the surface connecting with the occipital region, there is a screw feed mechanism. By turning this screw, the surface in contact with the occipital region can be adjusted manually in the range of 1.75 mm forward and backward to fit the shape of the head (Figure 2C), and because there is an attached rubber plate to prevent slipping, the equipment can be firmly secured to the head of the participant (Figure 3F). Additionally, it is possible to adjust the sensor position manually within the range of 10 mm, in order to match the size of the test subject’s head (Figure 2B), and using the C-shaped stainless steel elastic body (Figure 3D) linking the left and right temporal regions and the occipital region, the temporalis is clipped. By preventing shaking of the camera and mouthwitch, even when shaking one’s head, walking, or running, we are aiming for stable camera capture and measurement of temple movement. The results of verifying the clipping force (clip force f) in Figure 3D are as shown in Figure 5 for test results Section 4.1. The mass of the head-mounted equipment is 82 g. As the prototype in this paper is for evaluating the switch operation performance, it is not equipped with a camera, but it is also possible to load a compact, lightweight camera in the camera mount section shown in Figure 1.

From the mouthwitch block diagram in Figure 4, the temple sensor is mounted on the equipment shown in Figure 2, and a computer, speaker, and power device (omitted from Figure 3) are connected using the cable in Figure 1. The temple sensor has a compact optical sensor QRE1113 (Fairchild Semiconductor International Inc., San Jose, CA, USA) [23] attached. This optical sensor is equipped with one infrared LED (940 nm) and one phototransistor, and the LED is illuminated using infrared light near the temple. This reflected light is received by the phototransistor and measures the movement of the temple. More specifically, the temporal muscle extends and contracts, and the shape of the skin around the temple changes, altering the distance between the temple skin and temple sensor. The movement of the temporal muscle (mouth opening and closing) is measured from the changes in reflected light occurring due to changes in the positional relationship between the temple sensor and temple skin. The temple sensor is covered using a light-shielding cover to prevent excess environmental light (direct sunlight, etc.) from entering the phototransistor (Figure 1). The analog signal measured by the temple sensor undergoes AD conversion at a resolution of 12 bits with a sampling frequency of 10 Hz, using the Analog-Digital (AD) converter. The measurement values converted by the AD converter into digital signals are stored in memory. Data from the most recent 2-s interval are stored in memory using the First In, First Out (FIFO) system. Therefore, the measurement values recorded in memory consist of 20 items of voltage data *v*_i_ (*i* = 0, 1, 2, …, 19) measured by the temple sensor in intervals of 0.1 s.

The data *v*_i_ are classified by the classifier into TMC and other data, and in the case of TMC, the camera shutter is operated using the controller. The algorithm used by the classifier is discussed in the next section, Section 2.2. The prototype in this paper is not equipped with a camera, but when the classifier judges that TMC has occurred (shutter operation has occurred), a “Do (C)” sound is generated by the sound generator as a shutter sound, and this is sounded out by the speakers, communicating to the test subject that a shutter operation has occurred.

The timing display LED, in the verification tests described in Section 3, communicated the timing of TMC to the test subject, and consisted of one blue LED.

In this study, the five parts of the AD converter, timing communication LED, memory, sound generator, and classifier, are all realized in one microcomputer, the mbed LPC1768 (Switch Science Inc., Tokyo, Japan) [24], with in-house software (C language). Although not shown in Figure 1, a tablet terminal surfacePro3 (Microsoft Corp., Redmond, WA, USA) [25] was attached to the mbed NXP LPC1768 using a wired USB connection, and communication used the RS-232C standard, with the contents of memory saved simultaneously to the tablet terminal. For the communication software, we used the CoolTermWin Version 1.4.7 (Free ware) [26] free software.

### 2.2. Algorithm

Here, we explain the algorithm for the classifier. This algorithm classifies whether the test subject has performed TMC (action of opening mouth for one second, and then closing it again) or some other operation, and this result is output to the camera as a 1-bit shutter operation signal. The signal sent to the camera, when it is classified that the operation performed by the test subject is TMC, is 1, and in other cases it is 0. When the operation performed by the test subject is classified as TMC, the shutter sound generated by the sound generator in Figure 4 is output from the speakers. This prototype uses the “Do” sound as a shutter sound. Feedback is thus given to the test subject with this shutter sound as to whether the operation succeeded.

The classifier, every time data is measured (every 0.1 s), calculates the correlation coefficient between the ground truth recorded in advance in memory, and the data *v_i_^j^* (*i* = 0, 1, 2, …, 19) for the most recent 2-s interval measured by the sensor; when these calculation results are 0.9 or higher, the operation carried out by the test subject is classified as TMC. However, if the amplitude (difference between the maximum value and minimum value of the data for the most recent 2 s measured by the temple sensor) of the data *v_i_^j^* (*i* = 0, 1, 2, …, 19) for the most recent 2-s interval measured by the temple sensor is not in the range of 0.5*a* to 2.0*a* (the value *a* can be obtained through Equation (2)), this measurement can be excluded as noise. So, even if the correlation coefficient is 0.9 or higher, the operation carried out by the test subject shall not be classified as TMC. The ground truth *g_i_* (*i* = 0, 1, 2, …, 19) is obtained through the Equation (1) for each test subject. TMC was carried out 10 times (trials) for the test subject with the TMC data for each time (trial) expressed in the suffix *j* = 1, 2, 3, …, 10.

(1) $$\;g_{i}\;=\;\begin{array}{c} Me\\ j \end{array}\left(e_{i}^{j}\right),\;i\;=\;0,\;1,\;2,\;\ldots ,\;19,\;j\;=\;1,\;2,\;3,\;\ldots ,\;10\;$$

Here, $\begin{array}{c} Me\\ j \end{array}$ is the function for obtaining the median value of 10 items of data *e*_i_^1^, *e_i_*^2^, …, *e_i_*^10^. *e_i_^j^* (*i* = 0, 1, 2, …, 19) is obtained by normalizing the TMC measurement data set *v_i_^j^* (*i* = 0, 1, 2, …, 19) for the trial *j* with Equation (3).

(2) $$\;a\;=\;\begin{array}{c} Ave\\ j \end{array}\left(max_{j}-min_{j}\right),\;j\;=\;1,\;2,\;3,\;\ldots ,\;10\;$$

Here, $max_{j}$ and $min_{j}$ indicate the maximum and minimum values, respectively, for the measurement data set *v_i_^j^* (*i* = 0, 1, 2, …, 19) for TMC for one trial *j*. $\begin{array}{c} Ave\\ j \end{array}$ is a function for obtaining the mean value of 10 items of data *max*_1_ − *min*_1_, *max*_2_ − *min*_2_, …, *max*_10_ − *min*_10_.

(3) $$\;e_{i}^{j}\;=\;\frac{v_{i}^{j}-min_{j}}{max_{j}-min_{j}},\;i\;=\;0,\;1,\;2,\;\ldots ,\;19,\;j\;=\;1,\;2,\;3,\;\ldots ,\;10\;$$

For this algorithm, we applied the earable TEMPO algorithm [12] we developed previously. The earable TEMPO algorithm uses Equations (1) and (3). The mouthwitch also uses Equation (2) for noise exclusion.

## 3. Evaluation Tests

### 3.1. Test Subject

The test subjects were 8 healthy people (males and females between the ages of 21 and 44 years old, with a mean age of 24.5 years old), and these were referred to as A, B, C, D, E, F, G, and H, respectively. The test subjects consisted of people who had no self-recognition of pain in the mouth or head area, and those people who were receiving treatment for the mouth or head area were also excluded. This device can also place a camera in the line of vision of the study subjects and, because the camera is attached to the right-side of the head area on this device, test subjects whose right eye was stronger were used. 

The test subjects had a head of between 150 mm and 165 mm. The head-mounted equipment shown in Figure 2 and Figure 3 fit the head of the participant well without being either too big or too small. For each test subject, B and C in Figure 2 were adjusted so that the head-mounted equipment was fixed securely to the head of the participant, and the temple sensor was set so that it was positioned on the temple of the test subject. Test subjects for whom the head-mounted equipment did not match their heads, or those who felt uncomfortable due to the tightening (clamping force) of the head-mounted equipment, were excluded. 

This study was approved by the “Shinshu University ethics committee concerning research into humans”, and research cooperation was obtained after fully explaining the research to subjects in advance. Additionally, for all tests, the mouthwitch (equipment attached to the head), was cleaned with a brush and then washed and disinfected with ethanol disinfectant in consideration of hygiene issues.

### 3.2. Measuring the Clipping Force f of Head-Mounted Equipment

The head-mounted equipment shown in Figure 2 and Figure 3 measures the relationship between clipping force f of the temporal area and the head width. When the Head Length Adjustor *l*_1_ for the head-mounted equipment in Figure 5 is changed within 0 and 10 mm, and when the Head Width *l*_2_ is changed within 120 mm and 180 mm at 10 mm increments, Digital Force Gauge ZP-200N (IMADA Co., Ltd., Toyohashi, Japan) [27] is used as the Clipping Force *f*/2 N. Note that when the clipping Force *f*/2 is ON (at non-load), *l*_2_ is 115 mm.

### 3.3. Measurement of Data for Creating Ground Truth

In the state where the temple sensor is attached to the head of the test subject, the “operation in which the mouth was opened for 1 s and then closed (TMC)” was executed 110 times to match the timing in which the LED to indicate timing was lit up, and the value measured by the temple sensor at that time was recorded. This measurement was carried out indoors so that sunlight did not influence the temple sensor.

From the measurement results using the method in Section 2.2, ground truth was performed once for each test subject. The LED to indicate timing was repeated ten times with 1 s ON and 1 s OFF.

### 3.4. Mouthwitch Evaluation Test

This evaluation test evaluated performance when resting, speaking, chewing (chewing gum), walking, and running. The speaking test was carried out to investigate the effect that moving the mouth when speaking had on the mouthwitch operation (judgment of TMC). The gum chewing test was conducted to investigate the extent of the effect that such an action had on the judgment of TMC because the temporal muscles are also extended and contracted when chewing. The walking and running tests were conducted to investigate what effects body oscillations conducted for the purposes of walking or running have on the mouthwitch TMC judgment.

In this evaluation test, the test subjects rested for 180 s, then stated each of the alphabet letters from A, B, and C to Z once, chewed gum 100 times, walked for 80 s, and engaged in running for 80 s. Furthermore, in accordance with the lighting up of the timing indicator LED, the subjects carried out TMC 10 times each. For the gum chewing test, 3.0 g of gum (LOTTE Co., Ltd., Tokyo, Japan) [28] was chewed. The 80 s of walking involved the total time for 10 TMC of approximately 2 s each time, and 60 s spent not performing TMC. The timing indicator LED had the lighting timing set in accordance with the test content in advance. In the speaking test, when performing TMC, A, B, and C to Z were not vocalized, and these were spoken between TMC and TMC. Additionally, as in the gum chewing test, the chewing of gum was not performed during the time of TMC only and, during this time, the gum was kept in the subject’s mouth, with chewing of the gum only taking place between TMC and TMC.

In this test, the ground truth was set in advance in the memory of the test device in accordance with the test subject. Every time measurement was performed with the temple sensor (every 0.1 s), the correlation coefficient with ground truth was calculated, and when the correlation coefficient value was 0.9 or higher, the sound “Do (C)” was generated as the shutter sound, instead of operating the shutter, and played from the speakers.

The timing indicator LED lighting situation, the temple sensor measurement values, and the correlation coefficient between the measurement value and ground truth, were automatically recorded in the tablet terminal connected to the device in Figure 1. 

This measurement was carried out indoors so that environmental light, such as sunlight, did not influence the temple sensor.

### 3.5. Head-Mounted Equipment Evaluation Test

In these evaluation tests, we investigated the impact on the TMC measurement results of reattaching the mouthwitch and using it over a long period of time, to evaluate the head-mounted equipment. We asked test subject G to attach and remove the mouthwitch 20 times, and perform TMC once, matching the flashing of the timing display LED every time it is reattached. We recorded the correlation coefficient of the TMC measurement values and the ground truth over 20 times. Next, we asked test subject G to attach the mouthwitch and perform TMC 10 times, matching the flashing of the timing display LED. Following this, we asked the subject to watch a 60-min drama with the mouthwitch continuously attached. The drama that test subject G was asked to watch was the TV drama “Bewitched”, which was broadcast in America in 1964. After watching the drama, they were asked to perform TMC 10 more times, matching the flashing of the timing display LED. Then we recorded the correlation coefficient of the TMC measurement values and the ground truth over these 20 times.

## 4. Results

### 4.1. Results of Measuring Head-Mounted Equipment Clip Force f

**Figure 6:**
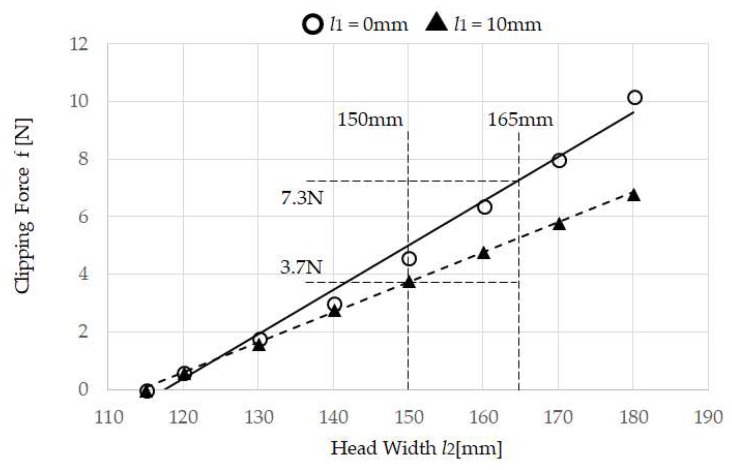
Measurement results of head-mounted equipment clip force *f*. As the head of the test subjects were between 150 mm and 165 mm, the scope of the clip force *f* of the head-mounted equipment in the evaluation tests in Section 4.2 was 5.5 ± 1.3 N (from 3.7 to 7.3 N).

Using the method in Section 3.2, the measurement results for the clip force *f* of the head-mounted equipment are shown in Figure 6. For the clip force *f*, double the value measured by Digital Force Gauge was obtained. As the head of the test subjects were between 150 mm and 165 mm, the scope of the clip force *f* in the evaluation results in Section 4.2 from Figure 6 was 5.5 ± 1.3 N (from 3.7 to 7.3 N).

### 4.2. Mouthwitch Evaluation Test Results

**Figure 7:**
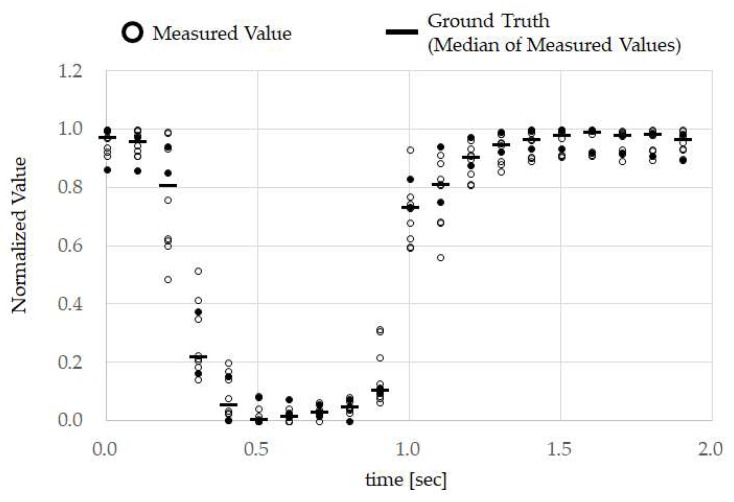
Test subject A’s ground truth. For ground truth, we had test subject A repeat TMC 10 times and, during that time, measured the movement of the temple skin using the temple sensor. The measurement values for these 10 times were respectively normalized, and the median values for each time were obtained and created from these normalized values. These measurement values were obtained using the method in Section 3.3.

**Figure 8:**
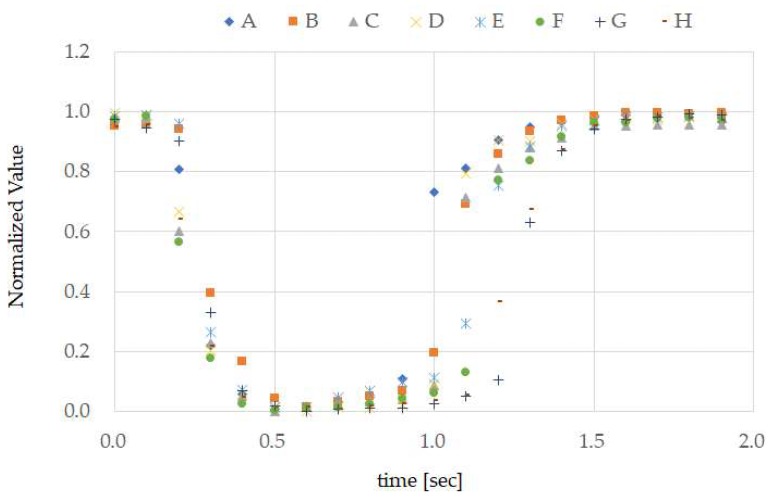
Ground truth of test subjects A to H. The ground truth was obtained for test subjects B to H using the same method as in Figure 7, and these results have been overlapped and displayed.

A section of the data used for creating ground truth measured according to the method in Section 3.3, and the ground truth obtained using the method of Section 3.3 are shown in Figure 7 and Figure 8, respectively. In Figure 7, the ground truth for test subject A and the temple sensor measurement results used in the creation of the ground truth are shown. For the ground truth, 20 data items measured by the temple sensor every 0.1 s are considered 1 set, and normalization was performed on each set. We calculated and created the median value for these normalized 10 sets of data for each time. There are methods that use the mean value rather than the median value, but mean values are known to be easily affected by outliers, and because median values are not so affected, and median values are considered to be stronger against noise than mean values, they were used. The ground truth for all test subjects is shown in Figure 8.

The mouthwitch evaluation results using the method from Section 3.4 are shown in Table 1. The *accuracy* in Table 1 was obtained using Equation (4). The closer the *accuracy* is to 1, the more correct the classification is in regard to whether the test subject performed TMC.

(4) $$\;accuracy\;=\;\frac{\mathrm{TP}\;+\;\mathrm{TN}}{\mathrm{TP}\;+\;\mathrm{FP}\;+\;\mathrm{FN}\;+\;\mathrm{TN}}\;$$

Here, TP (True Positive) refers to the number of times that the classifier judged TMC in cases where the subject actually performed TMC (correlation coefficient of 0.9 or higher), whereas the FP (False Positive) refers to the number of times that the classifier judged TMC despite the fact that the test subject did not actually perform TMC. TN (True Negative) refers to the number of times when the subject did not perform TMC and the classifier did not judge TMC (times when the correlation coefficient was less than 0.9), and FN (False Negative) was the number of times when, despite the fact that the test subjects performed TMC, the classifier did not judge TMC. Additionally, *precision* and *recall* in Table 1 were obtained using Equations (5) and (6), respectively.

(5) $$\;precision\;=\;\frac{\mathrm{TP}}{\mathrm{TP}\;+\;\mathrm{FP}\;}\;$$

(6) $$\;recall\;=\;\frac{\mathrm{TP}}{\mathrm{TP}\;+\;\mathrm{FN}\;}\;$$

Here, *precision* indicates the ratio when, among the cases where the classifier classified the test subject as having actually performed TMC, the test subject did in fact actually carry out TMC. *Recall* indicates the ratio when, among the subjects that actually performed TMC, the classifier classified the test subjects as having actually performed the TMC.

In Table 1, the mean values for *accuracy* in the 8 test subjects was 1.000 when resting, speaking, chewing, and walking, Similarly, the mean value for *precision* in all items was 1.000 and the mean value for *recall* was 1.000 when resting and chewing, 0.988 when speaking, 0.975 when walking, and 0.875 when running. 

**Figure 9:**
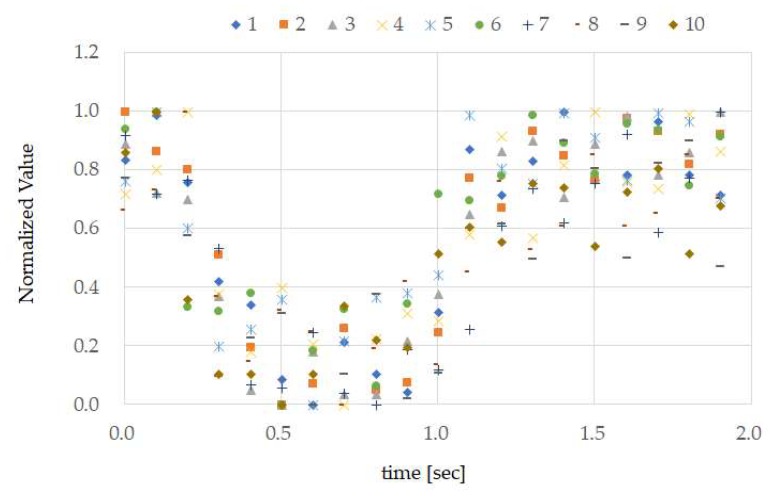
Measured values of subject B while running: The result was obtained by normalizing the measurement values of the temple sensor when person B performed the TMC 10 times while running. These measurement values were obtained from the experiment discussed in Section 3.4. While running, the measurement waveform for the temple sensor was distorted, and this led to a decrease in *accuracy* and *recall*.

Figure 9 shows the results of measured values of subject B while running. The result is obtained by normalizing the measurement values of the temple sensor when person B performs the TMC 10 times while running. These measurement values were obtained from the experiment discussed in Section 3.4. While running, the measurement waveform for the temple sensor was distorted, and this led to a decrease in *accuracy* and *recall*.

In the above tests, none of the test subjects bit their tongue by mistake when opening and closing their mouths. Additionally, none of the respondents actually practiced the TMC operation, but all were able to complete it swiftly. Furthermore, none of the subjects felt fatigue in the mouth and none felt a sense of discomfort due to tightening of the head-mounted equipment.

### 4.3. Head-Mounted Equipment Evaluation Test Results

**Figure 10:**
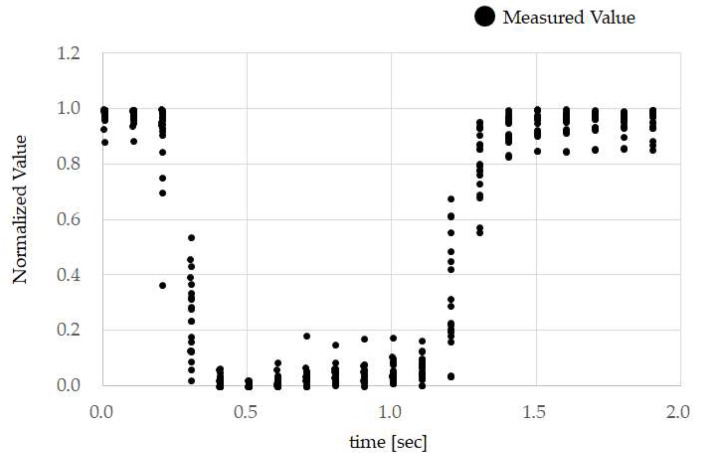
Measured values of subject G attaching and removing the device: this figure shows the results of test subject G attaching and removing the head-mounted equipment 20 times, and performing TMC for each reattachment, normalized based on Equation (3).

**Figure 11:**
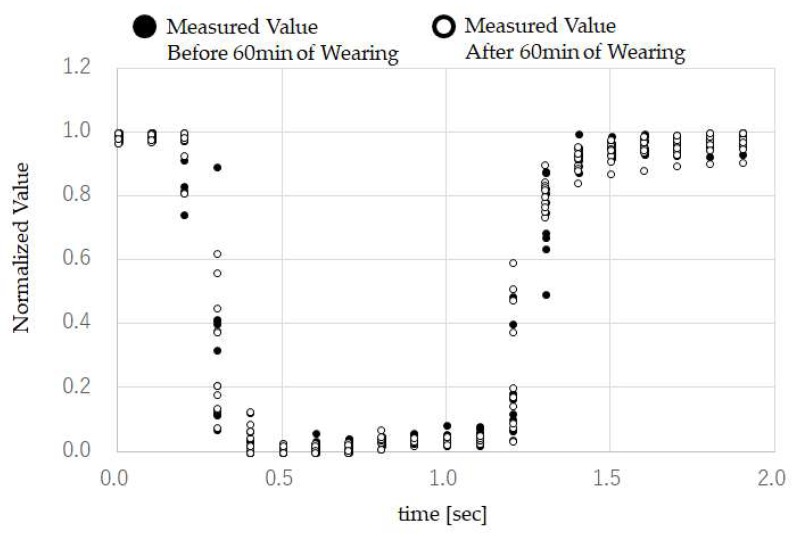
Measured values of subject G wearing the device for an hour: this figure shows the measurement values when test subject G performed TMC 10 times immediately after attaching the mouthwitch, and TMC 10 times again after 60 min of wearing it continuously, normalized based on Equation (3).

The evaluation test results for the head-mounted equipment based on the method in Section 3.5 are shown in Figure 10 and Figure 11. Figure 10 shows the results of test subject G attaching and removing the head-mounted equipment 20 times, and performing TMC for each reattachment, normalized based on Equation (3). The average value of the correlation coefficient over 20 times when performing reattachment was 0.977. Figure 11 shows the measurement values when the test subject performed TMC 10 times immediately after attaching the mouthwitch, and TMC 10 times again after 60 min of wearing it continuously, normalized based on Equation (3). The average value of the correlation coefficient for the 10 times before long-term usage was 0.985, and after use it was 0.982.

## 5. Discussion

From the results in Figure 7, it can be seen that, when test subject A opened their mouth, the normalized calculation values decreased, and when they closed their mouth, the values increased. In other words, we can also see that performing TMC made the calculation wave take on a “U” shape. Furthermore, from Figure 7, we can see that there was high reproducibility in TMC. From the ground truth in Figure 8, it is evident that the TMC calculation wave is “U”-shaped for other test subjects as well. Although not shown in this paper, there was also high reproducibility of TMC from test subjects B to H. The difference in ground truth with other test subjects shown in Figure 8 was the time during which the mouth was continually open. As, in this way, consistency was seen in the measurement results of the small number of 8 test subjects with different genders and ages, and sufficient investigation may be possible even with this number.

The mean values for *accuracy* when speaking and running from Table 1 were 0.002 lower than the comparative figures for other operations. The values are not thought to be at such a low level as to be problematic. As speaking and running are operations that are accompanied by the opening and closing of the mouth, the opening and closing of the mouth is a common point with TMC. It is thought that this common point influenced *accuracy*.

From the *precision* results in Table 1, we can see that there were no cases of mouthwitch mistakenly judging TMC even though the test subject had not performed TMC. Incidentally, one opens and closes one’s mouth when breathing while running; however, because this movement is different from opening one’s mouth for 1 s continuously and then closing it (TMC), mouthwitch does not mis-detect breathing while running for TMC.

As we can see from *recall* in Table 1, despite test subjects A, B, C, and D performing TMC while running, there were times when this was not classified as TMC by the classifier. Additionally, even though test subjects F and H performed TMC while speaking and walking, there were times when this was not classified as TMC by the classifier. These facts were observed using the results in Figure 8. If we focus on the normalized values of each test subject from 1.0 to 1.5 s on the time axis in Figure 8, we can see that, compared to test subjects A, B, C, and D, the time opening their mouth from 0.1 to around 0.3 s appears to be longer. Running, compared to speaking and walking, requires a faster breathing tempo and larger bodily movements (movements that are more frenetic within the unit time). In other words, when running, it is considered that the noise overlapping the temple sensors had a higher frequency compared to that when speaking and walking. Based on this, we can see that compared to movements with a high noise frequency, such as running, *recall* of low frequency TMC generated by lengthening the time that the mouth is opened has a better score. On the other hand, compared to slower movements, such as speaking and walking (movements with a low noise frequency), high frequency TMC generated by shortening the time the mouth is continually opened, and *recall* has a better score. As shown in Figure 9, the temple sensor measurement waveform is distorted when running. This is because, for the signal due to TMC, the amplitude of noise from breathing when running, and breathing and head-mounted equipment vibration when running, is too large to ignore. This high amplitude noise is linked to a reduction in *recall*. There is a method of increasing clip force f, as a means of preventing vibration in the head-mounted equipment. A method in which the area of the Non-Slip Rubber shown in Figure 3 is increased, and the material of the Non-Slip Rubber is changed to absorb more vibration, can be considered. As methods that increase the clipping force f raise the physical burden on the test subject, moving forward, we would like to proceed with a method of improving the Non-Slip Rubber. If we consider that mouthwitch will be used in everyday scenarios, this may not be used because photo blur may occur when taking pictures while running. Therefore, in addition to improving *recall* when running, it is also necessary to equip it with a function to track vibration and to adjust the shutter speed.

Based on the results of Figure 10, the correlation coefficient between ground truth and TMC was maintained, even when reattaching the mouthwitch. From the results of Figure 11, the correlation coefficient between ground truth and TMC was maintained, even after continuing to attach the mouthwitch continuously for 60 min. These results suggest that calibration is not required when reattaching the mouthwitch, or when using it over a long period of time. This also shows that the head-mount equipment was fixed securely to the head of test subject G.

Through verification tests, we can see that TMC has high reproducibility when resting, and this can be carried out rapidly, and performed simply and correctly without the test subject practicing.

These results suggest that TMC is suitable for movements where the objective is to operate a camera. Because mouthwitch can be integrated with headphones, it is highly compatible with devices that handle voice information, and because it does not obstruct your line of sight, it can be safely used in everyday life. For this reason, in addition to cameras, this can also be utilized for hands-free operation for devices, such as smartphones and music players, that are currently operated by hand. This may also lead to the development of wearable devices with new concepts using the mouthwitch. In the future, we would like to increase the number of temple sensors and place them on both the left and right temples, thus expanding the current 1-bit switch function to 2 bits.

Because the temple sensor uses an infrared sensor, sunlight that includes strong infrared waves is thought to affect the temple sensor measurement values. Moving forward, we need to survey this impact, and, where necessary, improve the temple sensor blocking cover, and provide environmental light filtering through signal processing, such as with modulation and demodulation technology. Additionally, since the user operates the shutter operation by opening and closing the mouth with mouthwitch, the gestures for this operation may cause those who do not know what mouthwitch is to feel that the TMC movements are strange. When looking at the user performing TMC, the subject may laugh, and this may make a good picture, but in a serious situation, the user may be mistaken for making light of the situation with the opening and closing of their mouth; therefore, when using mouthwitch, depending on the situation, it may be necessary for the user to cover their mouth with a mask. Additionally, so that the user easily understands that they can operate the shutter by opening and closing their mouth, it is necessary to raise awareness by using a signifier on the main unit of the mouthwitch, such as by displaying an icon [29,30,31]. In this way, in consideration of the actual operation of mouthwitch, it is also necessary for any proposal to consider customs and etiquette. By continuing to resolve the aforementioned issues and improving the tests and systems in a variety of environments, we aim to make mouthwitch a more practical solution.

## 6. Conclusions

We have developed a head-mounted camera operation interface (mouthwitch) in which the taking of pictures with a compact, lightweight head-mounted camera can be achieved, hands-free, by “opening your mouth continuously for approximately one second and then closing it again (TMC)”. Mouthwitch, using the originally developed temple sensor, optically measures changes in the shape of the temples that occur when the mouth is opened and closed. Based on these measurement results, the mouthwitch then judges whether this is TMC in which the user is intending to operate the camera shutter and, based on these measurement results, sends the shutter operation 1-bit control signal to the camera. The prototype in this paper is not equipped with a camera and, instead of this, when the prototype recognizes TMC, it outputs a “Do” noise, as a shutter noise, from the speakers.

We performed tests to evaluate the operations of mouthwitch in 8 subjects (males and females from 21 to 44 years old), when resting, speaking, chewing, walking and running. From these results, we confirmed that “opening your mouth continuously for approximately one second and then closing it again” could be performed reliably by all subjects, that the measurement results of shape changes that occur in the skin near the temples have high reproducibility, and that because they are performed swiftly, they are suitable for the camera shutter operation. Additionally, the mean values for *accuracy* across all 8 test subjects were 1.000 in case of resting, chewing, and walking, and 0.998 for speaking and running. At the same time, the mean *precision* values for all items was 1.000, and the *recall* mean values were 1.000 for resting and chewing, 0.988 for speaking, 0.975 for walking, and 0.875 for running. From the above results, we can see that, although there were times when the TMC performed by test subjects when running could not be recognized by the prototypes device, during the other items, TMC could be recognized with a high probability. Additionally, when the test subject could not perform TMC, this was not mis-detected by the prototype device as TMC.

Moving forward, we are aiming to improve *recall* when running and to improve the head-mounted equipment. Furthermore, we would like to consider customs and etiquette as matters requiring consideration when actually operating mouthwitch when making proposals. Additionally, we aim to improve tests and the system in a variety of environments and make mouthwitch a more practical solution.

## Figures and Tables

**Table 1:** Mouthwitch evaluation test results: For test subjects A to H, during 180 s of rest, the alphabet sounds from A to Z being spoken once, gum being chewed 100 times, walking for 80 s, and then, while running for 80 s, TMC was performed 10 times in accordance with the light indicator LED, and values for *accuracy*, *precision*, and *recall* were obtained from the test results. These values were the values obtained from tests using the method in Section 3.4 as well as Equations (4)–(6) in Section 4.2.

Subject	Item	*Accuracy*	*Precision*	*Recall*
A	Rest	1.00	1.00	1.00
	Speech	1.00	1.00	1.00
	Chewing	1.00	1.00	1.00
	Walk	1.00	1.00	1.00
	Run	1.00	1.00	0.90
B	Rest	1.00	1.00	1.00
	Speech	1.00	1.00	1.00
	Chewing	1.00	1.00	1.00
	Walk	1.00	1.00	1.00
	Run	0.99	1.00	0.50
C	Rest	1.00	1.00	1.00
	Speech	1.00	1.00	1.00
	Chewing	1.00	1.00	1.00
	Walk	1.00	1.00	1.00
	Run	1.00	1.00	0.90
D	Rest	1.00	1.00	1.00
	Speech	1.00	1.00	1.00
	Chewing	1.00	1.00	1.00
	Walk	1.00	1.00	1.00
	Run	0.99	1.00	0.70
E	Rest	1.00	1.00	1.00
	Speech	1.00	1.00	1.00
	Chewing	1.00	1.00	1.00
	Walk	1.00	1.00	1.00
	Run	1.00	1.00	1.00
F	Rest	1.00	1.00	1.00
	Speech	1.00	1.00	1.00
	Chewing	1.00	1.00	1.00
	Walk	1.00	1.00	0.90
	Run	1.00	1.00	1.00
G	Rest	1.00	1.00	1.00
	Speech	1.00	1.00	1.00
	Chewing	1.00	1.00	1.00
	Walk	1.00	1.00	1.00
	Run	1.00	1.00	1.00
H	Rest	1.00	1.00	1.00
	Speech	0.99	1.00	0.90
	Chewing	1.00	1.00	1.00
	Walk	1.00	1.00	0.90
	Run	1.00	1.00	1.00
Average	Rest	1.000	1.000	1.000
	Speech	0.998	1.000	0.988
	Chewing	1.000	1.000	1.000
	Walk	1.000	1.000	0.975
	Run	0.998	1.000	0.875
